# Extension of bacterial rDNA sequencing for simultaneous methylation detection and its application in microflora analysis

**DOI:** 10.1038/s41598-023-28706-w

**Published:** 2023-04-07

**Authors:** Motoi Nishimura, Tomoaki Tanaka, Syota Murata, Akiko Miyabe, Takayuki Ishige, Kenji Kawasaki, Masataka Yokoyama, Naoko Hashimoto, Kazuyuki Yamagata, Hidekazu Nagano, Satomi Tojo-Nishimura, Kazuyuki Matsushita

**Affiliations:** 1 Division of Laboratory Medicine, Clinical Genetics and Proteomics, Chiba University Hospital, Chiba, Japan; 2 Department of Molecular Diagnosis, Graduate School of Medicine, Chiba University, Chiba, Japan; 3 Research Institute of Disaster Medicine, Chiba University, Chiba, Japan

**Keywords:** Microbiology, Molecular biology

## Abstract

Although polymerase chain reaction (PCR) amplification and sequencing of the bacterial 16S rDNA region has numerous scientific applications, it does not provide DNA methylation information. Herein, we propose a simple extension for bisulfite sequencing to investigate 5-methylcytosine residues in the bacterial 16S rDNA region from clinical isolates or flora. Multiple displacement amplification without DNA denaturation was used to preferentially pre-amplify single-stranded bacterial DNA after bisulfite conversion. Following the pre-amplification, the 16S rDNA region was analyzed using nested bisulfite PCR and sequencing, enabling the simultaneous identification of DNA methylation status and sequence data. We used this approach (termed sm16S rDNA PCR/sequencing) to identify novel methylation sites and a methyltransferase (M. MmnI) in *Morganella morganii* and different methylation motifs among *Enterococcus faecalis* strains from small volumes of clinical specimens. Further, our analysis suggested that M. MmnI may be correlated to erythromycin resistance. Thus, sm16S rDNA PCR/sequencing is a useful extension method for analyzing the DNA methylation of 16S rDNA regions in a microflora, providing additional information not provided by conventional PCR. Given the relationship between DNA methylation status and drug resistance in bacteria, we believe this technique can be effectively applied in clinical sample testing.

## Introduction

Bacterial 16S rDNA (rRNA) gene sequencing has enabled the simple estimation of bacterial species using genetic testing and opened the door to microflora analysis. Although bacterial 16S rDNA sequencing analyzes a relatively small region and is generally limited by a lack of strict species-level identification accuracy^1^, it does not require bacterial culture and can estimate bacterial genus or species with fair accuracy. Combined with short-read next-generation sequencing (NGS), 16S rDNA gene amplicon-based analysis of bacterial communities is possible and relatively straightforward^2^. At present, 16S rDNA sequencing has been widely incorporated into various fields of science, including food analysis^3–5^, environmental surveys^6,7^, and clinical tests^8–11^.

Metagenomic analysis of bacterial DNA has further developed in recent years and has been combined with epigenomic analysis^12^, leading to the discovery of new methylation motifs and novel DNA methyltransferases^13–15^, while this new epigenomic technique is based on sequencing the whole genome and/or all nucleic acids in a sample. Additionally, random methylation analysis of DNA molecules in a sample does not necessarily provide the information to directly infer the species from which each sequence originates. Epigenomic studies on isolated and cultured bacteria have been conducted since before the advent of the new epigenomics^16,17^, and bisulfite-treated whole genomic DNA of the *E. coli* K-12 strain has been sequenced using epigenomic analysis^16^. N6-methyladenine (6 mA), 4-methylcytosine (4mC), and 5-methylcytosine (5mC) are frequently detected in bacteria, and accurate bisulfite-based sequencing techniques are available to measure 5mC^18^. Besides those in *E. coli*^16^, whole genome and targeted bisulfite sequencing have been used successfully to characterize m5C motifs in *Enterococcus faecalis*^19^ and *Prevotella intermedia*^17,20^. A ten-eleven translocation (Tet) protein-assisted bisulfite sequencing method has also been developed to extend the bisulfite sequence to allow the detection of 4mC^21^. Single-molecule real-time (SMRT) sequencing from Pacific Biosciences was introduced to the study of 6 mA and 4mC methyltransferases when this technology was reported in 2010^22^. Since then, the SMRT sequencing has been a fundamental technology for the successful detection of 6 mA and 4mC and methylome analysis of various bacterial species including *Leptospira interrogans*^23^, *Clostridioides difficile*^24^, and *Pseudomonas aeruginosa*^25^. In contrast to that of 6 mA and 4mC, the SMRT sequencing kinetic signal associated with 5mC is significantly weaker. A recently published epigenomics method describes the use of methylated-cytosine selective restriction enzymes^17^; unlike previous methods, the new epigenomic analyses^12^ does not require a single-molecule sequencer and does not change the large-scale analysis of non-targeted, random DNA molecules.

Performing DNA methylation analysis completely in parallel with conventional 16S rDNA sequencing analysis may provide DNA methylation data by targeting information-containing regions, efficiently leading to the identification of bacterial genus or species. Thus, we hypothesized that the combination of 16S rDNA sequencing and bisulfite polymerase chain reaction (PCR)^26^ can provide additional DNA methylation information while retaining the existing benefits of 16S rDNA analysis. Furthermore, since 16S rRNA has several hairpin structures^27^, and palindromic sequence can form hairpin structures, 16S rDNA may contain palindromic nucleotide sequences. Most prokaryotic DNA methyltransferases recognize palindromic DNA sequences^28^; thus, 16S rDNA may be a region for detecting DNA methylation.

Despite the limitations^1^, 16S rDNA sequencing of isolates is performed as a regular clinical test in clinical microbiology laboratories and is now spreading worldwide^29^. In other words, in many countries, 16S sequence information on clinical isolates from specimens are available in these laboratories routinely. Therefore, additional bisulfite sequencing of the 16S region, even if only partially, is a cost-effective and accurate way to analyze DNA methylation in this region. The computational resources required for this analysis may be sufficient with a computer of similar performance to those used for the routine testing. In contrast, although whole genome epigenetic analysis combined with whole genome sequencing provides a vast amount of accurate and comprehensive information, this is not routinely performed as a clinical test worldwide. Therefore, not only additional sequencing costs and hands-on time, but also additional costs for computer resources are required. Clinical laboratories may be unable to afford such costs. The advantages and limitations of adding bisulfite sequencing to the 16S rDNA sequencing in isolates, as described so far, can be extended to microflora analysis. Comprehensive methylome analysis of a microflora enables accurate species identification and total understanding of DNA methylation of the flora, but it requires very large sequencing power and the added cost of analyzing big data. DNA methylation analysis of the 16S rDNA region of microflora is not as rigorous or comprehensive as comprehensive methylome analysis, but it is simple and its moderate size can be handled by a relatively inexpensive short-read desktop NGS, and, above all, 16S rDNA is a parameter that clinical laboratories are familiar with analyzing.

The degree of methylation and the number of methylation sites on bacterial DNA differ greatly between species^30^. Therefore, in this study we designed primer sequences for the bisulfite PCR used in the combined analysis, including a degenerated universal primer set suitable for detecting the variable methylation content in the 16S rDNA region. This universal primer set can target both bisulfite-treated and non-bisulfite-treated bacterial genomic DNA; therefore, 16S rDNA bisulfite PCR/sequencing described here can be performed in parallel with non-bisulfite-treated 16S rDNA PCR/sequencing using the same primer set to provide *simultaneous* information on the methylation status of the targeted 16S rDNA in addition to the sequence itself. Thus, we designated the combination of these PCR and sequencing methods sm16S rDNA PCR/sequencing.

The purpose of this study is to propose sm16S rDNA sequencing, an extension of the widely-used 16S rDNA sequencing, and demonstrate its potential applications, such as in the discovery of novel methylation motifs and DNA methyltransferases using clinical microflora specimens and cultured isolates.

## Results and discussion

### Improvement of conditions for 16S rDNA bisulfite PCR ; pretreatment using MDA without DNA denaturation (MDAwoDD)

Bisulfite PCR in the 16S rDNA region and sequencing of the PCR products require some considerations. One reason for this is that bisulfite treatment denatures the target DNA, damaging it and making it single-stranded. Bisulfite treatment for methylcytosine-selective hydrolysis causes a significant level of target degradation^31^. Target degradation can be a potential drag on PCR amplification. Even if the target DNA is not damaged, single-stranded DNA (ssDNA) is less amplified in PCR than double-stranded DNA (dsDNA) (Supplementary Fig. S1). Thus, in 16S rDNA bisulfite PCR, treated target ssDNA is at risk of being less amplified than non-denatured contaminated dsDNA. Further, the possibility of bacterial contamination from the lab environment cannot be entirely ignored, especially because contaminating bacteria typically have double-stranded 16S rDNA, which can skew our observations. For example, it is known that commercially available PCR reagents and DNA extraction kits may be contaminated with bacterial DNA, which may interfere with the results of experiments such as sequencing^32–34^. PCR reagent contamination, such as DNA polymerase contamination, can cause mixing of unintended bacterial DNA at the start of PCR.

To reduce the contamination risk and repair degraded DNA, as a pretreatment for bisulfite PCR of the 16S rDNA region, we performed multiple displacement amplification (MDA) on template DNA handled in neutral conditions (Supplementary Fig. S1a) to avoid its denaturation, which is usually performed on DNA denatured in alkaline conditions. The reason why alkaline-treated DNA is used in MDA is because double-stranded DNA cannot be primed by oligomers in the MDA reaction^35^ (thus conventional MDA requires single-stranding of the template DNA). This is advantageous since MDA without DNA denaturation (designated MDAwoDD) preferentially amplifies ssDNA in a sample (Supplementary Fig. S1b and S1c).

### MDAwoDD successfully amplifies bisulfite-converted DNA

To perform bisulfite PCR coupled with MDAwoDD, we extracted genomic DNA from DNA cytosine methyltransferase (dcm)+ and dcm− *E. coli* strains. Next, DNA was treated with bisulfite and amplified with MDAwoDD. Untreated DNA was amplified with conventional MDA as a control. The MDA product was digested with the restriction enzymes MluCI (NEB, Ipswich, MA, USA) and Sau3AI (Takara Bio, Kyoto, Japan) to determine whether the bisulfite-treated DNA was amplified. MluCI recognizes AATT, whereas Sau3AI recognizes GATC sites. Bisulfite treatment converts unmethylated cytosine to uracil. Therefore, in the amplified product from bisulfite-treated DNA, many of the Sau3AI recognition sites will disappear, whereas the number of MluCI recognition sites will increase. Compared with the undigested sample, the MDA product showed little change when digested with Sau3AI but was entirely digested with MluCI (Supplementary Fig. S2a). This result indicates that almost all the amplified DNA was derived from bisulfite-converted DNA and that amplification from an unconverted pool was negligible.

The MDA products amplified using the DNA extracted from a clinical isolate (*Klebsiella oxytoca*) or using the DNA extracted from a clinical microflora sample (urine-derived) showed the same trend of being less digestible by Sau3AI and more digestible by MluCI (Supplementary Fig. S2b, S2c), suggesting that the converted DNA was amplified similarly to the previously tested *E. coli* genomic DNA.

### Design of Bisulfite PCR targeting 16S rDNA region and primer sets

The 16S rDNA V4-V5 region was the target region for bisulfite PCR coupled with MDAwoDD. The V4 region is not only a target for commercial 16S rDNA analysis kits^36^, for example a target for NEXTflex™ 16S V4 Amplicon-Seq Kit 2.0 for Illumina Library Prep (Perkin Elmer, Waltham, MA, US), but also convenient for testing the ability to detect 5-methylcytosine. For example, in *E. coli*, the V4-V5 region contains five dcm methylation sites (CC(A/T)GG), as determined from the NCBI NC_000913 genomic sequence. This region was used because the target region size could be designed to fit into the read length of a short-read next-generation sequencer, such as the Thermo Fisher Scientific Ion PGM sequencer. Indeed, the V4-V5 region is also targeted by a commercial 16S rDNA analysis kit (16S V4-V5 Library Preparation Kit, Norgen Biotek Corporation, Thorold, ON, Canada).

**Figure 1:**
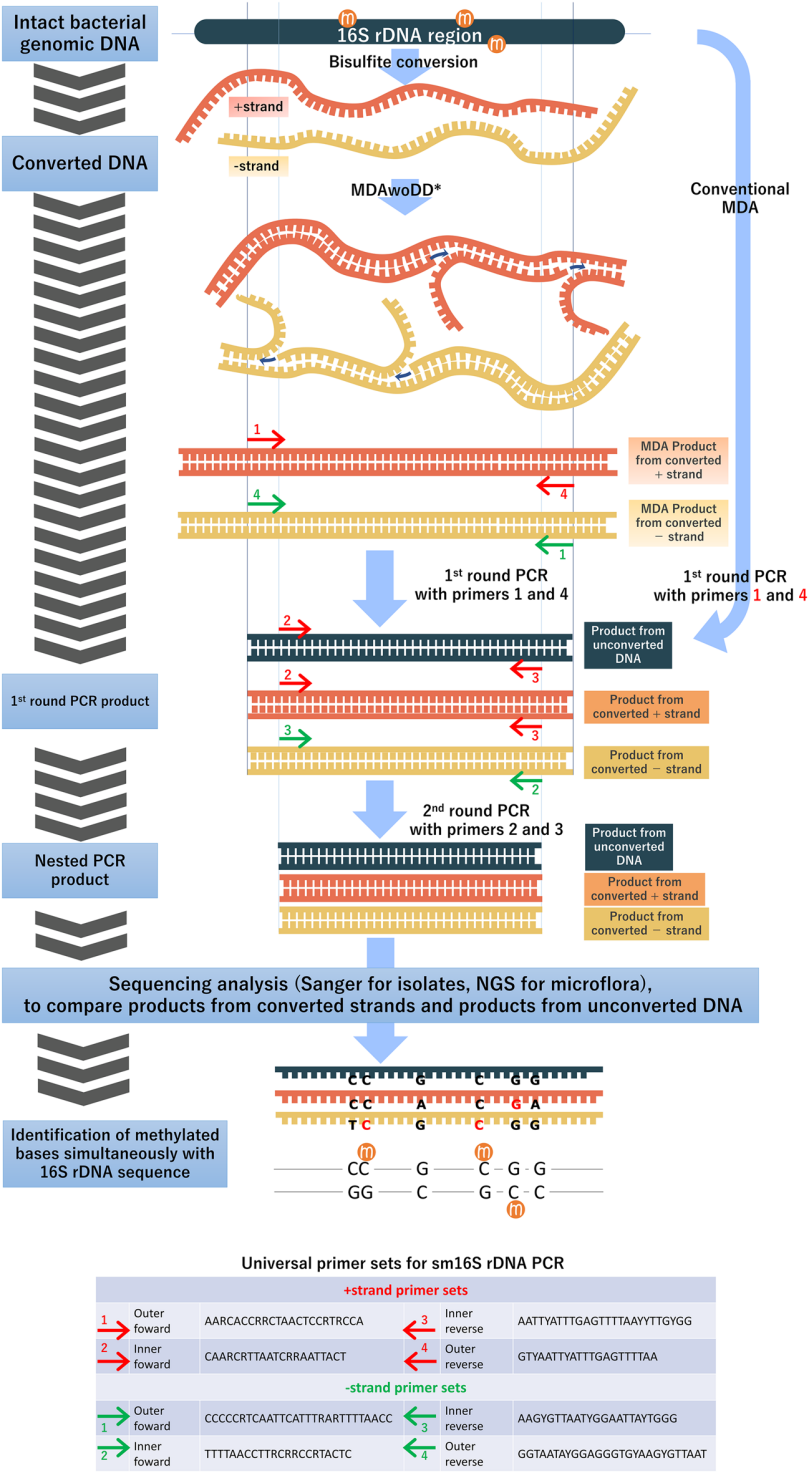
sm16S rDNA PCR and primer sets. The bacterial 16S rDNA V4-V5 region was targeted using bisulfite PCR. Universal nested PCR primer sets were designed to amplify both bisulfite-converted and non-bisulfite-converted DNA. The primer sites were chosen to cover several bacterial species and reduce degenerate nucleotides. *MDA without DNA denaturation (MDAwoDD) was used for the amplification and repair of bisulfite-converted DNA. When amplifying unconverted DNA, the conventional MDA method was used. MDA; multiple displacement amplification. R; A or G. Y; C or T. “m” within the orange circle indicates a methylcytosine base.

To increase the specificity of bisulfite PCR for this region, a universal primer set for nested PCR was designed for each of the positive-sense (+) and negative-sense (−) strands (Fig. 1). The bisulfite PCR products were analyzed using benchtop NGS (Ion PGM) or Sanger sequencing (Materials and methods).

### sm16S rDNA PCR method has better cycle threshold (Ct) values than conventional method

To quantitatively compare the 16S rDNA bisulfite PCR step in sm16S rDNA PCR with the conventional bisulfite PCR, a TaqMan assay was performed in the 2nd round PCR of the nested PCR described above. In this bisulfite PCR step in sm16S rDNA PCR, 500 pg of genomic DNA from NEB 5-alpha *E. coli* was bisulfite-converted and pre-treated with MDAwoDD, followed by the nested PCR. For the conventional bisulfite PCR, the same amount of bisulfite-converted NEB 5-alpha *E. coli* genomic DNA was amplified directly by nested PCR without pretreatment such as MDA, using the primers shown in Fig. 1.

Ct value of bisulfite PCR step in sm16S rDNA PCR, indicated as mean ± standard deviation, was 23.34 ± 1.61, while that of the conventional method was 31.24 ± 0.83 (n = 12, p < 0.000001, Welch's t-test). This means that our proposed sm16S rDNA PCR has better Ct values than that of the conventional bisulfite PCR. Thus, sm16S rDNA PCR may have a potential for clear amplification with smaller PCR cycles compared to conventional bisulfite PCR.

### sm16S rDNA PCR from clinical isolates and microflora

**Figure 2:**
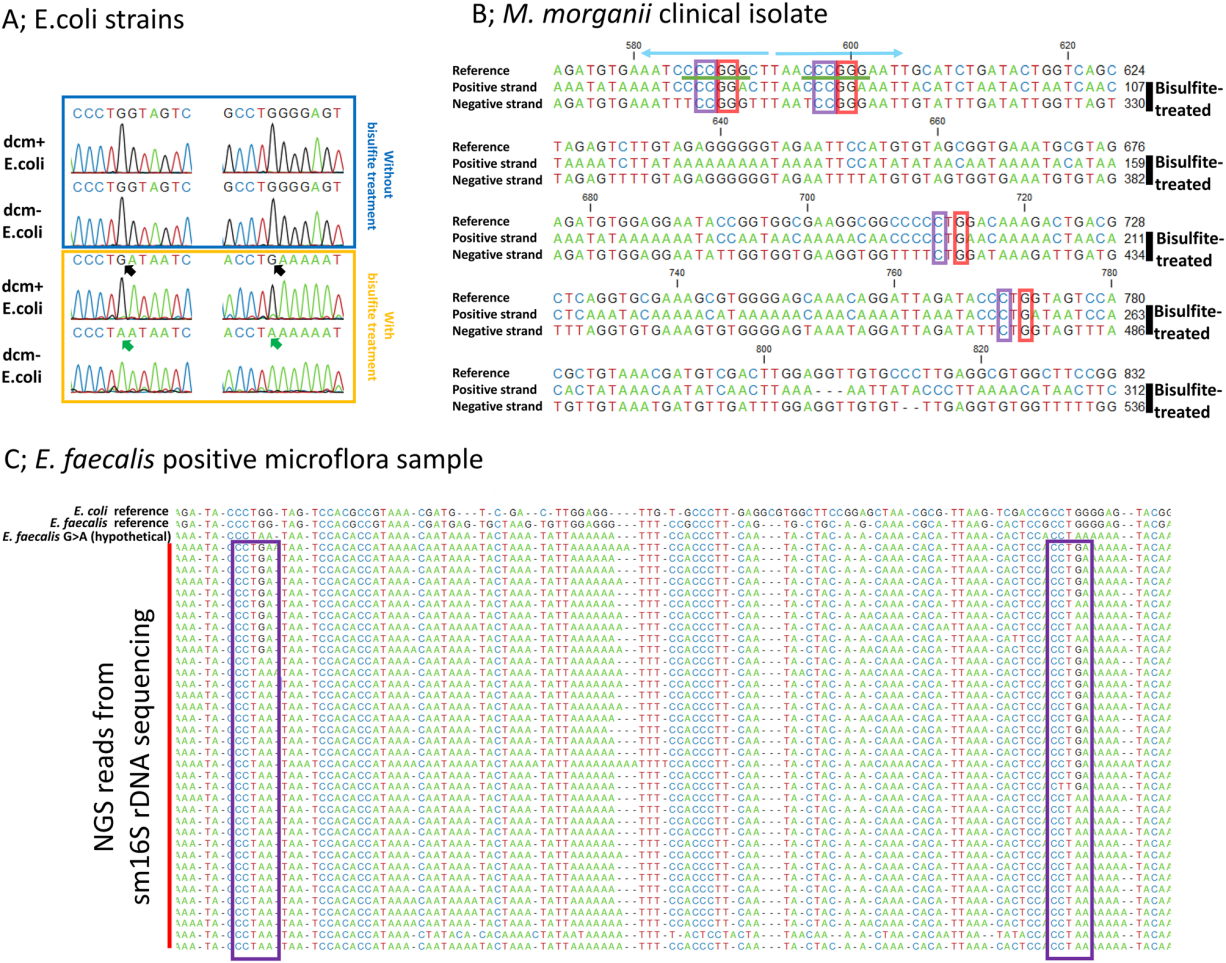
Sequencing the sm16S rDNA PCR product from clinical isolates and microflora simultaneously provides rDNA sequence information and methylation status. Extracted DNA was converted with bisulfite treatment and amplified with MDAwoDD. DNA without bisulfite treatment was amplified using conventional MDA. Bisulfite PCR was performed using MDA products as templates, and the resulting PCR products were sequenced. (**a**) Sanger sequencing of bisulfite PCR products from genomic DNA of dcm+ and dcm− *E. coli*. The left side of the figure shows a typical CC(A/T)GG site targeted by dcm methylase and the right half shows another typical CC(A/T)GG site. The difference between dcm+ and dcm− *E. coli* becomes apparent with bisulfite treatment, as shown by the black and green arrows. Green arrows indicate G to A conversion resulting from bisulfite conversion and unmethylated cytosine in the negative-sense strand. The black arrow (not converted) indicates the existence of 5-methylcytosine in the negative-sense strand. (**b**) Summary of bisulfite sequencing from a clinical isolate of *M. morganii*. The red boxes indicate 5-methylcytosine in the negative-sense strand. The purple boxes indicate 5-methylcytosine in the positive-sense strand. Note that the four methylated residues at the two SmaI sites (CCCGGG) are underlined in green. The four methylation residues exist in a palindrome-like structure, as indicated by the light blue arrows. The reference sequence was retrieved from NCBI NR_028938.1. c) Sequence reads implicating diversity in genomic DNA methylation status of *E. faecalis* from a microflora sample. Sequencing of amplicons from MDAwoDD products indicated diverse DNA methylation at the CC(A/T)GG site (purple boxes) of the genomic DNA fragment that appears to be from *E. faecalis* in sample number 10_1379 (Supplementary Table 1). In the purple boxes, the Gs written in black in the bisulfite-converted sequences indicate 5-methylcytosine in the negative-sense strand. The reference sequences in the top two columns were retrieved from NCBI NC_000913 (*E. coli*) and NCBI NR_114782.1 (*E. faecalis*) and are the intact sequences, not the bisulfite-converted ones. *E. faecalis* G>A (hypothetical) sequence in the third column from the top was generated by simply substituting nucleotides, assuming that all cytosines are unmethylated and subject to bisulfite conversion.

Next, the 16S rDNA bisulfite PCR step of sm16S rDNA PCR was performed on the amplified MDA products at neutral pH using a nested primer set (Fig. 1), and the obtained products were sequenced. The difference in methylation between dcm+ and dcm− strains was detected at all five dcm methylation sequences [CC(A/T)GG] in the *E. coli* 16S rDNA V4-V5 region (Fig. 2a). Thus, sm16S rDNA sequencing was able to detect the cytosines methylated by dcm methylase in the target region.

Genomic DNA from clinical isolates were bisulfite-converted and then used as a template for MDAwoDD and for testing the downstream product (Supplementary Fig. S2b). The MDAwoDD product was subjected to the nested PCR and sequencing. In genomic DNA from *K. oxytoca*, methylation of CC(A/T)GG sites was detected (Supplementary Fig. S3a), similar to sites in dcm+ *E. coli*, as previously reported^37^. Besides *K. oxytoca*, genomic DNA was extracted from clinical isolates of several species, including *Proteus mirabilis*, *Pseudomonas aeruginosa*, and *Staphylococcus epidermidis*, and was subjected to sm16S rDNA sequencing. Within the target region, 5-methylcytosine was not detected in *Proteus mirabilis* (Supplementary Fig. S3b). We observed partial methylation in *Pseudomonas aeruginosa* and *S. epidermidis* (Supplementary Fig. S3c, S3d). As shown so far, sm16S rDNA sequencing was able to detect DNA methylation in a variety of bacterial species. For *Morganella morganii*, we observed methylation at the CC(A/T)GG sequence and four methylated residues at two consecutive SmaI sites that were encompassed by a palindrome-like structure (Fig. 2b). As shown in Supplementary Fig. S3c and d, a partial methylation pattern was detected in both gram-negative (*Pseudomonas aeruginosa*) and gram-positive (*S. epidermidis*) bacteria. Partial methylation in bacterial genomes was reported in several epigenetic studies^16,38^. These studies reported that partial DNA methylation may have some significance in relation to bacterial growth^16^. Such cases are among the targets for future verification and further research.

Clinical microflora DNA was successfully amplified with MDAwoDD (Supplementary Fig. S2c), and sm16S rDNA PCR and sequencing were performed on the MDA products. DNA extracted from the bacterial flora in urine samples was used as the clinical microflora sample. These clinical samples were simultaneously subjected to isolation culture and routine bacterial identification tests. As in the clinical isolate experiment, the results of non-bisulfite converted sequence in the sm16S rDNA sequencing were consistent with those of clinical bacterial identification tests by isolation culture and matrix-assisted laser desorption/ionization-time of flight (MALDI-TOF) mass spectrometry (Supplementary Table S1). However, the findings were not in perfect agreement because clinical bacterial identification tests are performed on specimens that have been isolated and routinely-cultured and MALDI-TOF–MS is used for rapid bacterial identification at the species level^39^. The results of bacterial identification between PCR-based and culture-based identification conducted on clinical specimens generally agree with each other; however, discrepancies are often noted^40,41^. It is also presumed that one factor contributing to this discrepancy is that routine culture from urine has low sensitivity for bacterial identification and requires special efforts^42^.

In a previous study, 16S rDNA analysis of urinary microflora showed that the number of species or genera of bacteria detected in the urine of each patient ranged from 1 to 7^43^. In our analysis of urine samples, we also observed a slightly higher number of bacterial species or genera of bacteria per patient than that in the previous study. However, our findings also indicated the possibility of diversity in the methylation status of the genomic DNA fragment that appears to be from *Enterococcus faecalis* in microflora sample number 10_1379 (Fig. 2c). An examination of other samples has yielded data implying that DNA may be methylated in *E. coli*, and the DNA methylation pattern seen may be identical to that seen with the well-known dcm methylase (Supplementary Fig. S4.).

Although constitutive genomic DNA methylation at the GCWGC and CCGG sites of an *E. faecalis* isolate has been previously reported^19^, methylation at the CC(A/T)GG site, as detected in this study, has not been previously reported. It is difficult to determine from the data shown in Fig. 2c whether the diversity of methylation at the CC(A/T)GG site is due to the mixing of *E. faecalis* strains with different constitutive genomic DNA methylation. The data can be interpreted to reflect how methylation at the CC(A/T)GG site varies from cell to cell, even in the same strain. Additionally, it is possible that genomic DNA fragments from unknown or known different bacterial species with the same sequence as *E. faecalis* were mixed and analyzed (Fig. 2c). Therefore, we conducted an experiment using clinical isolates and NBRC (Biological Resource Center, NITE) bank strains of *E. faecalis* to verify whether there is a diversity in the stable DNA methylation in *E. faecalis*. Indeed, the genomic DNA methylation at the CC(A/T)GG site in *E. faecalis* was constitutively different among isolates and strains (Supplementary Fig. S5); interestingly, Supplementary Fig. S5 shows that the constitutive DNA methylation pattern at the CC(A/T)GG site in *E. faecalis* may not only include the well-known pattern (CmCWGG) seen, for example, in *E. coli* dcm methylase, but also atypical patterns that occur at both ends of the CCWGG site (mCCWGG), depending on the *E. faecalis* strain. Therefore, we cannot rule out the possibility that the diversity of DNA methylation seen in Fig. 2c is still due to mixing of *E. faecalis* strains with different constitutive genomic DNA methylation.

### A novel *M. morganii* enzyme is responsible for the consecutive methylation

In *M. morganii*, four methylated residues at two consecutive SmaI sites within a palindrome-like structure were detected, suggesting the presence of an uncharacterized DNA cytosine-methyltransferase (Fig. 2b). Since DNA methylation within such palindrome-like sequences has functional implications other than the restriction-modification system^44^, we searched for the enzyme responsible for the consecutive methylation.

**Figure 3:**
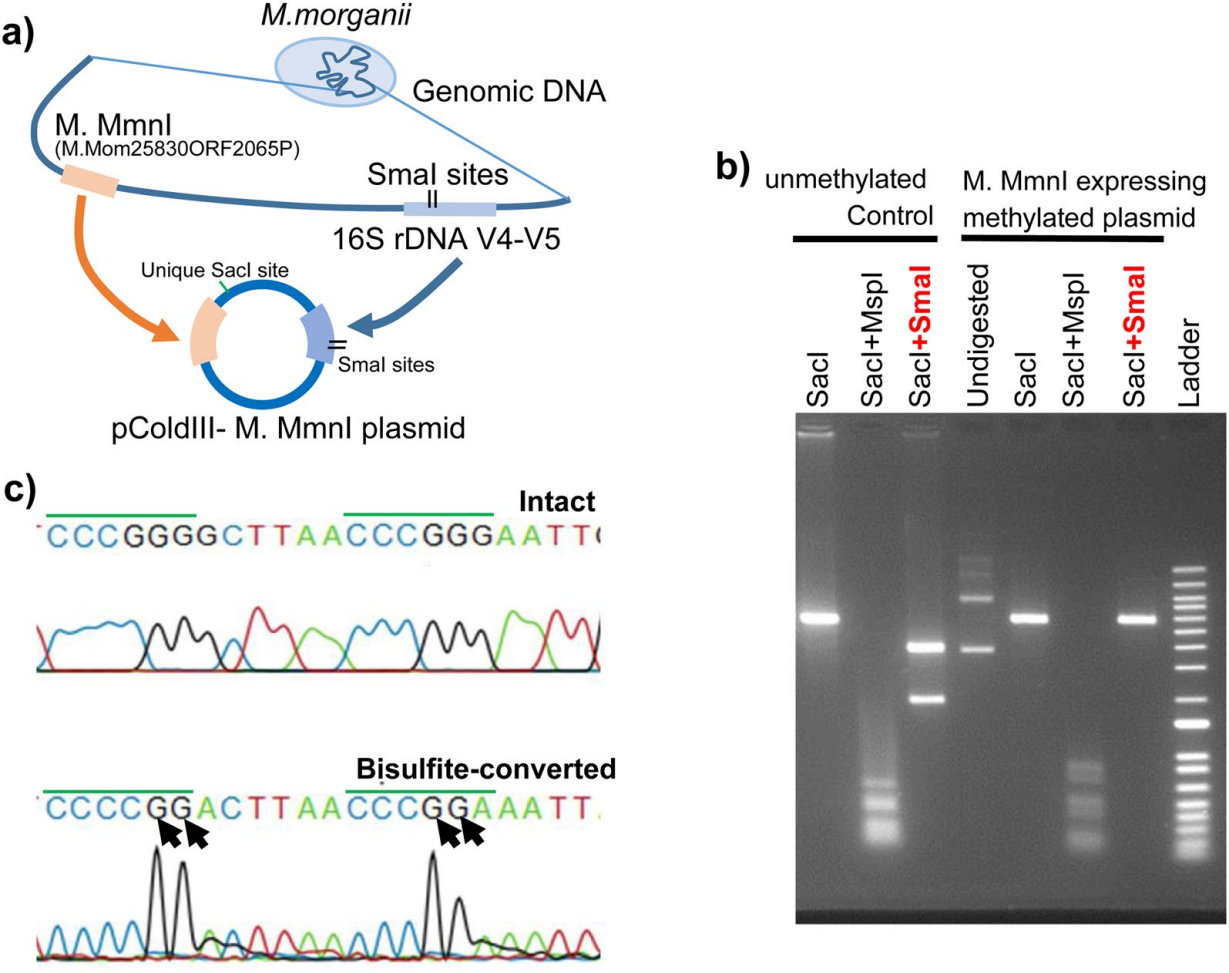
M. MmnI is responsible for four methylated residues at two consecutive SmaI sites within a palindrome-like structure. (**a**) Graphical scheme of pColdIII-M. MmnI (renamed from M.Mom25830ORF2065P) plasmid construction. (**b**) Restriction digestion of the pColdIII-M. MmnI plasmid evaluated via agarose gel electrophoresis. MspI and SmaI were used, where the plasmid contained 37 CCGG and two CCCGGG target sites. Plasmid DNA was linearized using a unique SacI site. Thermo Fisher E-Gel 1 kb plus ladder was employed as a size marker. Control unmethylated DNA was prepared using a Repli-g kit (QIAGEN). (**c**) Bisulfite sequencing of the palindrome-like structure confirmed the presence of four methylated residues in the two consecutive SmaI sites within the structure. The two SmaI sites are overlined in green. Black arrows indicate 5-methylcytosine in the negative-sense strand.

M.Mom25830ORF6305P and M.Mom25830ORF2065P, which are presumed to encode DNA methyltransferases in *M. morganii*, were identified using the REBASE database. Next, these genes were each cloned into pCold III expression plasmids along with the palindrome-like structure. These constructs were expressed in the *E. coli* dcm− strain using a previously described method^12^ (Fig. 3a). The results showed that in M.Mom25830ORF6305P, digestion of the extracted plasmid with SmaI was possible and methylation within the palindrome-like structure did not occur. In M.Mom25830ORF2065P, digestion with SmaI was blocked, indicating methylation of the palindrome-like structure (Fig. 3b). Bisulfite sequencing of the palindrome-like structure confirmed the consecutive methylation (Fig. 3c). The product of the M.Mom25830ORF2065P gene was designated as methyltransferase MmnI (M. MmnI).

Although several DNA methylation patterns are already known at the SmaI site in previous studies, similar to M. MmnI, SpyI methylase (M. SpyI) causes consecutive methylation with 5-methylcytosine^45^. M. SpyI recognizes CCAGG, CCTGG, CCCGG, and CCGGG sequences, resulting in consecutive methylation at the SmaI site^45^. Similarly, in the plasmid encoding M. MmnI, methylation was found at these four sequences, suggesting that M. MmnI recognizes these four sequences and that there is partial homology between the amino acid sequences of M. MmnI and M. SpyI (Supplementary Fig. S5). Based on these results, we concluded that M. MmnI is the enzyme responsible for the four methylated residues at the two consecutive SmaI sites within the palindrome-like structure.

Since M. SpyI is associated with erythromycin (EM) resistance^45^, we investigated whether M. MmnI is also related to the responsiveness of *M. morganii* to EM^35^. The transformed *E. coli* dcm− strain containing the M. MmnI expression plasmid was estimated to have a slightly higher IC_50_ (50% growth-inhibitory concentration, approximately 70 mg/L) for EM than that of the *E. coli* dcm− strain with the plasmid lacking the M. MmnI open reading frame (approximately 32 mg/L) (Supplementary Fig. S6). *M. morganii* is naturally resistant to EM^46^. M. SpyI is associated with EM-resistant *S. pyogenes* strains. However, in *S. pyogenes* strains, EM resistance has been primarily explained by other drug resistance genes genetically linked to M. SpyI^45^. The knockout of dcm DNA methyltransferase, which recognizes CCAGG and CCTGG sequences, suppresses *E. coli* growth at lower EM concentrations^47^. Therefore, 5mC methyltransferases that recognize CCAGG and CCTGG sequences may be correlating factors, if not the main factors, in EM resistance.

## Conclusion

The sm16S rDNA PCR and sequencing method presented in this paper allows the simultaneous combination analysis of non-bisulfite converted DNA and bisulfite-converted DNA in the 16S rDNA region via a universal bisulfite PCR primer set. Thus, analysis of various bacteria (Fig. 2a, b, and Supplementary Fig. S3) and bacterial microflora can be performed using small-volume clinical samples. In fact, the sm16S rDNA PCR, which involves template DNA repair by MDAwoDD and restoration to double-stranded DNA, can achieve clear amplification with fewer cycles (Ct value) in the bisulfite PCR step. Although unintentional contamination of bacterial DNA from commercial PCR reagents may occur in this step^32–34^, amplification by sm16S rDNA PCR seems to be less affected to contamination than that by conventional bisulfite PCR, which uses single-stranded templates. Obtaining DNA methylation information in the 16S rDNA region is an efficient way to identify the bacteria, from which the methylation originates. Since sm16S rDNA sequencing can simultaneously infer species and identify DNA methylation patterns, our results allowed us to infer that *E. faecalis* has various methylation patterns depending on the strain or isolate (Fig. 2c).

Sequencing of 16S rDNA has been practically incorporated into clinical testing. In clinical laboratories, 50,000 bacterial isolation, culture, and identification tests are conducted annually at our hospital alone, and countless specimens are discarded after testing worldwide. The method reported here can be added on to the conventional 16S rDNA identification test and enables DNA methylation analysis of various bacterial species in these discarded specimens. Notably, these bacteria were alive at the time of disposal, which means that if DNA methylation is detected using this method (Fig. 2b), then live bacteria can be recovered and prepared for further analysis (Fig. 3). In addition, we found that M MmnI from *M. morganii* methylates four consecutive SmaI sites in the palindrome-like sequence within the 16S rDNA region (Figs. 2b and 3) and that M. MmnI may be correlated to EM resistance. The relationship between bacterial responses to antibiotics and DNA methylation status^48^ is an important subject for future analysis by sm16S rDNA sequencing. Further, our method may facilitate the detection of unknown DNA methylation patterns and the identification of the responsible methyltransferases. However, our method detects 5-methylcytosine (5mC) but not methyladenine (6 mA) or 4-methylcytosine (4mC), thus making it difficult to identify a type of methyltransferases that add methyl groups to produce 6 mA and 4mC. Following the approach of 4mC-TAB-seq, which enables detection of m4C by incorporating Tet protein treatment as a preprocessing step in bisulfite sequencing^21^, we plan to combine a similar preprocessing step with sm16S rDNA sequencing to enable the detection of m4C and m5C in our future research.

In clinical laboratories, sm16S rDNA PCR/sequencing may contribute to the elucidation of the relationship between DNA methylation and clinically valuable information such as drug resistance.

## Materials and methods

### Bacterial strains, clinical specimens, and DNA extraction

*Escherichia coli* DNA adenine methyltransferase (dam)+/dcm+ (NEB 5-alpha competent *E. coli*, #C2987) and dam−/dcm− (dam−/dcm− competent *E. coli*, #C2925) strains were purchased from NEB (Ipswich, MA, USA) and cultured in Luria–Bertani (LB) medium. Clinical isolates, namely *K. oxytoca*, *M. morganii*, *Proteus mirabilis*, *Pseudomonas aeruginosa*, *S. epidermidis*, *Serratia liquefaciens*, and *E. faecalis*, were cultured on agar plates for routine bacterial identification using MALDI-TOF mass spectrometry (Biotyper, Bruker Daltonics GmbH, Leipzig, Germany), at Chiba University Hospital^39^. *Enterococcus faecalis* NBRC strains were purchased from the National Institute of Technology and Evaluation (Tokyo, Japan). Bacterial flora from patient urine samples was washed twice with phosphate-buffered saline and separated via centrifugation at 3000×*g* for 5 min at a temperature of 20–25 °C. Each patient underwent routine clinical bacteriuria tests in which bacterial species were cultured on agar plates and identified using a Biotyper instrument. Bacterial DNA was extracted from either culture medium or separated urinary microflora using an innuPREP Bacteria DNA kit (Analytik Jena GmbH, Jena, Germany) according to the manufacturer’s protocol.

### 16S rDNA Bisulfite PCR coupled with MDAwoDD

Approximately half of the extracted DNA was quantified with Nanodrop (Thermo Fisher Scientific, Waltham, MA, USA) and subjected to bisulfite treatment using an innuCONVERT Bisulfite Basic kit (Analytik Jena). In the case of purchased *E. coli* strains and clinical isolates, approximately 200 ng of DNA was added to the bisulfite treatment as starting DNA. Microfloral DNA extracted from urine was from the crude fraction by centrifugation, and 1–2 µg of DNA was extracted from 1 mL of urine, of which 400–1000 ng of DNA was used for the bisulfite treatment. Approximately 25 ng (isolates) or 50 ng (microflora) from the remaining unconverted DNA was amplified with conventional MDA using a TruePrime WGA Kit (4basebio, Madrid, Spain). Analogously, the bisulfite-converted DNA, equivalent to 25 ng (isolates) or 50 ng (microflora) of starting DNA, was amplified with MDA without DNA denaturation (MDAwoDD) using the same kit. Both conventional MDA and MDAwoDD usually yielded approximately 10–25 µg of DNA per sample, which was confirmed by measuring with Nanodrop.

In conventional MDA, template double-stranded DNA is usually denatured with alkaline solution before the acid addition and amplification. However, in this study, the bisulfite-converted single-stranded DNA was not treated under alkaline conditions and amplified by neutralizing the alkaline solution with an acidic solution prior to DNA input (MDAwoDD). In other words, in the conventional MDA, double-stranded DNA is denatured to single-stranded DNA by adding the reagents in the order of template DNA, alkaline solution, and acidic solution. In MDAwoDD, equal amounts of the same reagents are used, but they are added in the order of alkaline solution, acid solution, and template DNA to prevent denaturation of DNA. MDA products from bisulfite-converted or unconverted bacterial DNA were subjected to nested PCR with the primer sets shown in Fig. 1. The universal primer sets for sm16S rDNA PCR were designed using the ApE (A plasmid Editor, https://jorgensen.biology.utah.edu/wayned/ape/) and Primer 3 (http://bioinfo.ut.ee/primer3-0.4.0/) software under the following five conditions: i) targeting the V4–V5 region of 16S rDNA; ii) relatively few degenerate nucleotides (R (A, G) or Y (C, T)); iii) primer length > 20 nucleotides; iv) prospects of priming more bacterial species, as expected from the results of BLAST targeting 16S rDNA sites; and v) an expected product size of 364 bp and 377 bp for *E. coli*, which is within the read length of the Ion PGM sequencer.

Bisulfite PCR step in sm16S rDNA PCR was performed using the primer sets and nested PCR (Fig. 1) utilizing KOD-Multi & Epi-DNA polymerase (TOYOBO, Tokyo, Japan) at a final concentration of 0.02 U/μL. For this bisulfite PCR, 1 µl of a tenfold diluted MDA product was used as the template in both cases, irrespective of whether it was an MDAwoDD product or a conventional MDA product. This volume of the template was equivalent to approximately 50–500 pg of starting DNA. In the nested PCR, the final primer concentrations were 0.6 μM in both the first and second PCR rounds. For the second PCR round, 2 µl of a tenfold dilution of the product of the first PCR round was used as a template. A universal primer set in the 1st round of the nested PCR is a combination of 5′-AARCACCRRCTAACTCCRTRCCA-3′ and 5′-GTYAATTYATTTGAGTTTTAA-3′ for amplification from MDAwoDD product of bisulfite-converted positive strand, and in the subsequent 2nd round PCR, a universal primer set combining 5′-CAARCRTTAATCRRAATTACT-3′ and 5′-AATTYATTTGAGTTTTAAYYTTGYGG-3′ is used (Fig. 1). For amplification from MDAwoDD product of bisulfite-converted negative strand, a universal primer set in the 1st round of the nested PCR is a combination of 5′-CCCCCRTCAATTCATTTRARTTTTAACC-3′ and 5′-GGTAATAYGGAGGGTGYAAGYGTTAAT-3′, and in the subsequent 2nd round PCR, a universal primer set combining 5′-TTTTAACCTTRCRRCCRTACTC-3′ and 5′-AAGYGTTAATYGGAATTAYTGGG-3′ is used (Fig. 1). The universal primer sets for the converted positive-strand (the primer sets for the converted negative-strand as well) were also designed to be used for the nested PCR from the conventional MDA product of unconverted bacterial DNA (Fig. 1). The cycling conditions were as follows: 94 °C for 2 min and 15 cycles (first round) or 20 cycles (second round) at 98 °C for 10 s, Tm °C for 30 s, and 68 °C for 15 s; Tm °C was 56 °C for the positive strand in the first PCR round, 42 °C for the negative strand in the first PCR round, and 52 °C for the second PCR round. Between the first and second rounds, excessive primers were removed with an enzymatic protocol using exonuclease I (NEB) and quick CIP (NEB).

### Comparison of cycle threshold (Ct) values between bisulfite PCR step in sm16S rDNA PCR and conventional bisulfite PCR using TaqMan assay

To quantitatively compare bisulfite PCR step in our proposed sm16S rDNA PCR with the conventional bisulfite PCR, a TaqMan assay was performed in the 2nd PCR of the nested PCR described above.

The 2nd PCR conditions in the TaqMan assay were as follows: 95 °C for 20 s and 40 cycles at 95 °C for 10 s and 58 °C for 25 s using StepOnePlus Real-Time PCR Systems (Thermo Fisher Scientific). PCR primers and the TaqMan probe used were follows; 5′-TTTTAACCTTRCRRCCRTACTC-3′, 5′-AAGYGTTAATYGGAATTAYTGGG-3′ (Fig. 1) and 5′ [FAM]-CCCCCCTCTACAAAACTCAAACTTACC-[TAMRA] 3′. Ct values were calculated using StepOne and StepOnePlus v2.3 software (Thermo Fisher Scientific).

### Sanger sequencing and NGS

Sanger sequencing was performed using a 3500xL Genetic Analyzer (Thermo Fisher Scientific). A BigDye Terminator v3.1 Cycle Sequencing Kit (Thermo Fisher Scientific) was used for the cycle sequencing reaction. NGS was performed using an Ion Plus Fragment Library Kit, a Hi-Q View OT2 Kit, a Hi-Q View Sequencing Kit, or an Ion 318 Chip Kit v2 (Thermo Fisher Scientific) and a benchtop Ion PGM system, according to the manufacturer’s protocol. The DNA fragmentation step was skipped when using the Ion Plus Fragment Library Kit.

In the case of analysis of purchased or clinical isolates, Sanger sequencing is performed to obtain two types of data: bisulfite-converted sequence data and non-bisulfite-converted data (Fig. 1).

From the sequence obtained from the non-bisulfite-converted data, a hypothetical sequence is generated by simply substituting nucleotides, assuming that all cytosines are unmethylated and subject to bisulfite conversion. Thereafter, using CLC Genomics Workbench (Qiagen, CLC bio, Aarhus, Denmark), the bisulfite-converted sequence data, the non-bisulfite-converted sequence, and the assumed sequence are compared to identify the bases that are resistant to bisulfite conversion and to detect cytosine methylation.

The sequence from non-bisulfite-converted data were checked for no significant deviations from the clinical testing information by running BLAST against the NCBI database (rRNA/ITS databases (16S ribosomal RNA sequences (Bacteria and Archaea))).

In the case of the urine microflora analysis, NGS sequencing was performed by Ion Torrent PGM, which still provided bisulfite-converted and non-bisulfite-converted sequence data. Both sequence data were then analyzed in an integrated manner using CLC Genomics Workbench (Qiagen). First, CLC Genomics Workbench (Qiagen) was loaded with the 16S RefSeq Nucleotide sequence record database downloaded from NCBI Genbank. The sequence data obtained from Ion PGM was then imported into CLC Genomics Workbench (Qiagen), primer sequences were trimmed, and BLAST analysis was performed against the loaded 16S sequence database to identify the genus or species of the bacteria included in the microflora. The bacterial species (or genus) with the highest number of reads identified were selected, for example, the top 20, and from these reads, as in the case of isolate analysis, hypothetical bisulfite-converted sequences were generated, assuming that all cytosines are unmethylated. Using these hypothetical sequences as a reference database, BLAST comparisons with experimentally obtained bisulfite-converted sequence data were performed using CLC Genomics Workbench (Qiagen) to identify cytosine methylation at the single nucleotide level of resolution.

### Plasmid preparation

To determine the DNA methyltransferase gene responsible for the consecutive methylation in the *M. morganii* 16S rDNA region (Fig. 2b), we selected M.Mom25830ORF6305P and M.Mom25830ORF2065P (designated as M. MmnI in this paper) as candidate genes by searching against a gold-standard dataset in the Restriction Enzyme Database (NEB REBASE)^49^. The genes were cloned into the pCold III–Mor1 expression vector between the SacI (Takara Bio) and XbaI (Takara Bio) restriction sites. For the M.Mom25830ORF6305P-expressing plasmid, the gene-specific oligonucleotide primers used were 5′-GGTGAACGGTTCAGACGACT-3′ and 5′-CCTGCGCTACTGTTTCGGTA-3′ in the first round of nested PCR and 5′-ATATGGAGCTCATGAAAAACACTGTTAATTT-3′ and 5′-TACCTATCTAGATCACGTGAAACTTTCAAGACC-3′ in the second round of PCR. For the M.Mom25830ORF2065P-expressing plasmid (designated pCold III-M. MmnI, Fig. 3a), the gene-specific oligonucleotide primers used were 5′-TGTTTTTCCGGCCTTCCTGT-3′ and 5′-CATCGGATTTTCAGCCGCTG-3′ in the first round of nested PCR and 5′-CATATGGAGCTCATGATTTTGAAAAAACACCC-3′ and 5′-TACCTATCTAGATTATTTTACCGGCGGTATTG-3′ in the second round of PCR. The entire cloned gene fragments in both plasmids were Sanger-sequenced.

The pCold III–Mor1 expression vector was constructed from pCold III vector (Takara Bio, Kyoto, Japan) at the NgoMIV (NEB) site by inserting the *M. morganii* 16S rDNA V4–V5 region sequence with the following additional NgoMIV site: 5′-GCCGGCCAAGCGTTAATCGGAATTACTGGGCGTAAAGCGCACGCAGGCGGTTGATTGAGTCAGATGTGAAATCCCCGGGCTTAACCCGGGAATTGCATCTGATACTGGTCAGCTAGAGTCTTGTAGAGGGGGGTAGAATTCCATGTGTAGCGGTGAAATGCGTAGAGATGTGGAGGAATACCGGTGGCGAAGGCGGCCCCCTGGACAAAGACTGACGCTCAGGTGCGAAAGCGTGGGGAGCAAACAGGATTAGATACCCTGGTAGTCCACGCTGTAAACGATGTCGACTTGGAGGTTGTGCCCTTGAGGCGTGGCTTCCGGAGCTAACGCGTTAAGTCGACCGCCTGGGGAGTACGGCCGCAAGGTTAAAACTCAAATGAATTGCCGGC-3′.

### Experimental verification of DNA methyltransferase activity

To verify the function of the M. MmnI gene, the plasmids were transformed into *E. coli* dam+/dcm+ (NEB 5-alpha competent *E. coli,* #C2987) and dam−/dcm− (dam−/dcm− competent *E. coli*, #C2925) strains. The *E. coli* strains were cultured in LB broth (Thermo Fisher Scientific) supplemented with 100 mg/L ampicillin (FUJIFILM Wako Pure Chemical Corporation, Osaka, Japan). Plasmid DNA was isolated using the FastGene Xpress Plasmid PLUS Kit (Nippon Genetics, Tokyo, Japan). SacI was used to linearize the pCold III-M. MmnI plasmid DNA, which was then inactivated by heat at 70 °C. The methylation status was assayed via enzymatic digestion using DNA-methylation-sensitive SmaI (NEB) and DNA-methylation-insensitive MspI (NEB) restriction enzymes (Fig. 3b). Unmethylated pCold III-M. MmnI plasmid DNA was prepared by amplification using a Repli-g mini kit (Qiagen). We further verified the methylated motifs of M. MmnI. Briefly, half of the pCold III-M. MmnI plasmid DNA extracted from the transformed *E. coli* dam−/dcm− cells was subjected to bisulfite conversion. Both converted and unconverted DNA were subjected to restriction digestion followed by agarose gel electrophoresis (Fig. 3b) and to Sanger sequencing as described above (Fig. 3c).

### Statistical analysis

All statistical analyses, including calculation of standard deviations and Welch's t-test, were performed using Excel 2016 (Microsoft, Redmond, WA, USA) with the add-in software Statcel 3 (OMS Publishing Inc., Saitama, Japan).

### Ethics

The present study design, including the associated consent forms and procedures (according to the Ethics Guidelines for Medical Research for Humans in Japan), was approved by the Human Ethics Committee of Chiba University (No. 685). Urine samples were obtained from patients who provided written informed consent, and the samples were then irreversibly anonymized according to the requirements of the ethics committee.

## Supplementary Information

Supplementary Information.

## Data Availability

The sm16S rDNA NGS sequencing data that supported this study have been deposited in the DDBJ DRA database (https://www.ddbj.nig.ac.jp/dra/index-e.html) under the accession numbers DRA013560, DRA013562, DRA013563, and DRA013564. DRA013560 supports Fig. 2c, whereas DRA013562 (from sample number 10_1417), DRA013563 (from sample number 10_1439) and DRA013564 (from sample number 10_1379) support Supplementary Table S1.
