# Supplementary material for: Extension of bacterial rDNA sequencing for simultaneous methylation detection and its application in microflora analysis
Source: Sci Rep. 2023 Apr 7;13:5731. doi: 10.1038/s41598-023-28706-w (PMC10082018; doi:10.1038/s41598-023-28706-w)
Supplement: Supplementary file 1 — Supplementary Information. [file 41598_2023_28706_MOESM1_ESM.docx]

**Supplementary Materials**

**Supplementary Table S1**.

**BLAST search results from non-bisulfite-converted sequences obtained by sm16S rDNA sequencing of microbiome samples are consistent with those of bacterial identification tests by isolation culture and MALDI-TOF mass spectrometry.**

| Clinical biome Sample No. | BLAST search result of 16S rDNA  bisulfite PCR sequencing of MDA  products from DNA without bisulfite treatment | Result of routine bacterial identification test by MALDI-TOF-MS at Chiba University Hospital |
| --- | --- | --- |
| 10_1379 | *Lacticaseibacillus songhuajiangensis*  *Delftia rhizosphaerae*  *Enterococcus* spp*.*  *Methylobacillus methanolivorans*  *Pseudomonas monteilii*  *Bacillus velezensis*  *Acinetobacter bereziniae*  *Bacillus solisilvae* | *Enterococcus faecalis* |
| 10_1417 | *Corynebacterium jeikeium*  *Delftia rhizosphaerae*  *Phenylobacterium* spp.  *Pseudomonas monteilii*  *Aliidiomarina soli*  *Morganella morganii*  *Caballeronia telluris*  *Nitrosospira tenuis*  *Orbus hercynius* | None detected* |
| 10_1439 | *Escherichia coli*  *Aliidiomarina soli*  *Cronobacter* spp*.*  *Pseudomonas* spp*.*  *Franconibacter pulveris*  *Phascolarctobacterium succinatutens*  *Xenorhabdus stockiae*  *Chromohalobacter israelensis*  *Staphylococcus* spp. | *Escherichia coli*  *Pseudomonas aeruginosa*  *Staphylococcus aureus*  *Corynebacterium striatum* |

DNA was extracted from precipitated bacteria in centrifuged urinary samples of three patients. Conventional MDA products from the untreated DNA were subjected to sm16S rDNA bisulfite sequencing using Sanger sequencing or NGS. BLAST search results for sequences with the top 10 read numbers are listed. When multiple species belonging to the same genus were identified, they were grouped together as species (spp.). Because MDA and PCR can also amplify DNA from dead bacteria, this approach can detect more species than routine bacterial identification tests based on live bacteria. *No colonies appeared in the isolation culture for routine bacterial identification test. MS, mass spectrometry.

**Supplementary Figures**

**
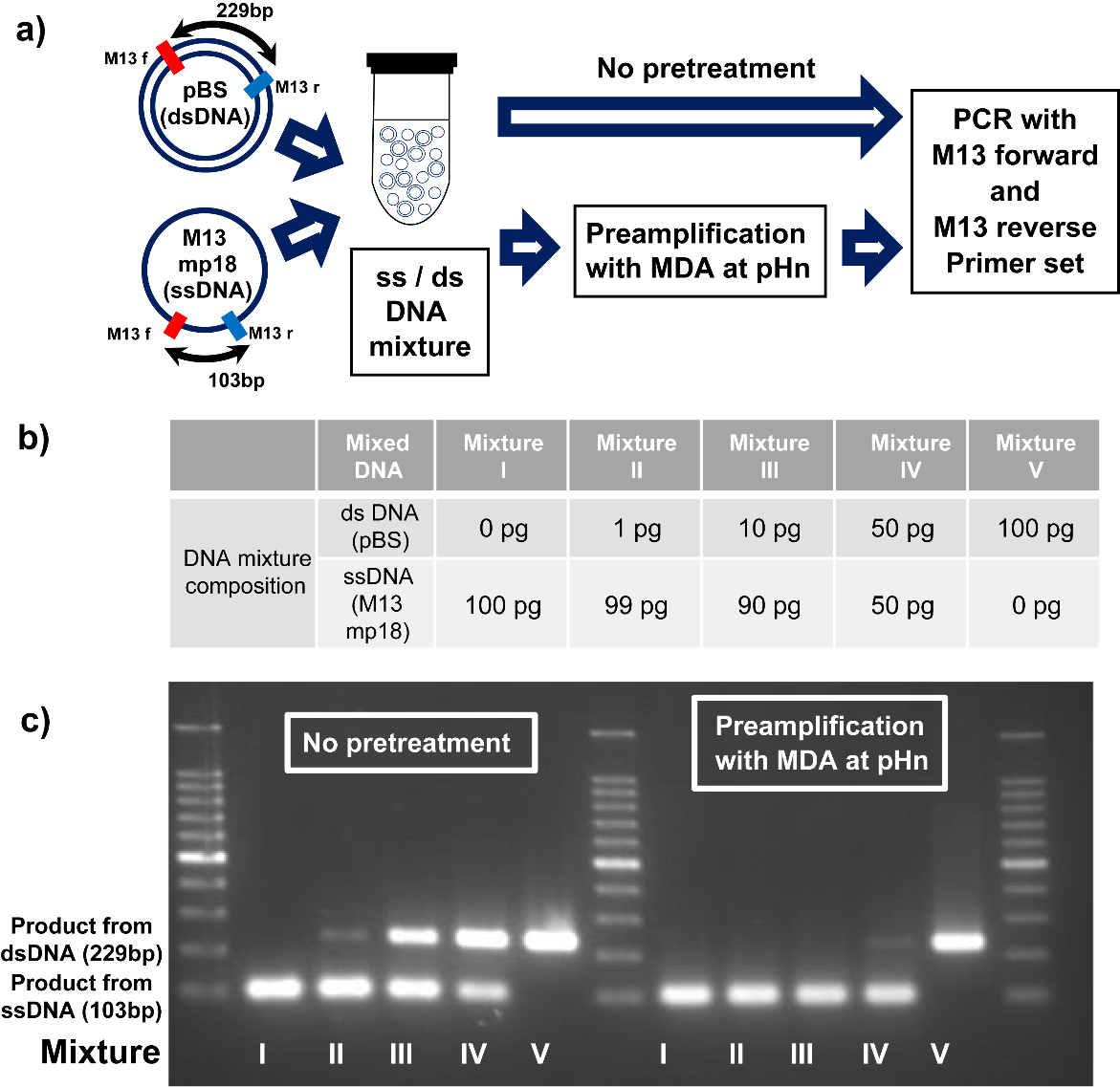
**

**Supplementary Figure S1**.

**Preamplification using MDA without DNA denaturation (MDAwoDD) has single-stranded DNA selectivity**

(a) Single-stranded (ss) M13 mp18 DNA and double-stranded (ds) pBS (pBluescript) DNA were mixed and used directly (no pretreatment) or indirectly (preamplification using MDAwoDD) as the PCR template using M13 forward and reverse primers. Both M13 mp18 and pBS contained M13 forward (M13 f, indicated in red) and M13 reverse (M13 r, indicated in blue) primer sites. However, the expected PCR product sizes were different (pBS 229 bp and M13 mp18 103 bp). Mixtures I–V had the compositions shown in (b). (c) Direct (no pretreatment) or indirect (preamplification using MDAwoDD) PCR results. When a DNA mixture of ssDNA and dsDNA was directly used as a template, dsDNA was preferentially amplified over ssDNA (mixture IV). When the DNA mixture was pre-amplified, ssDNA was preferentially amplified over dsDNA (in mixtures II–IV).

**An unprocessed version of the gel image of Supplementary Figure S1c**


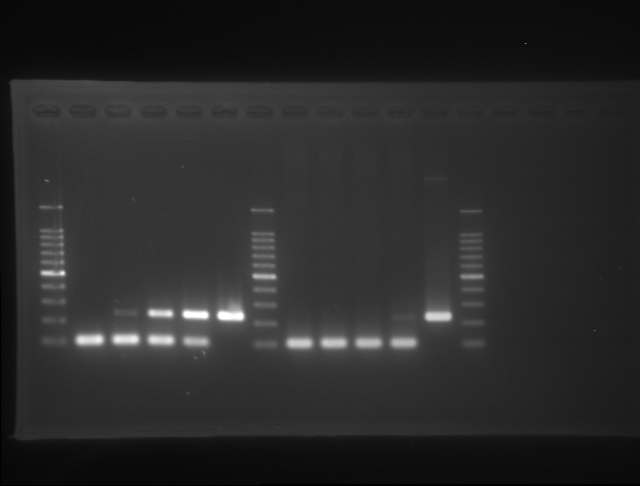


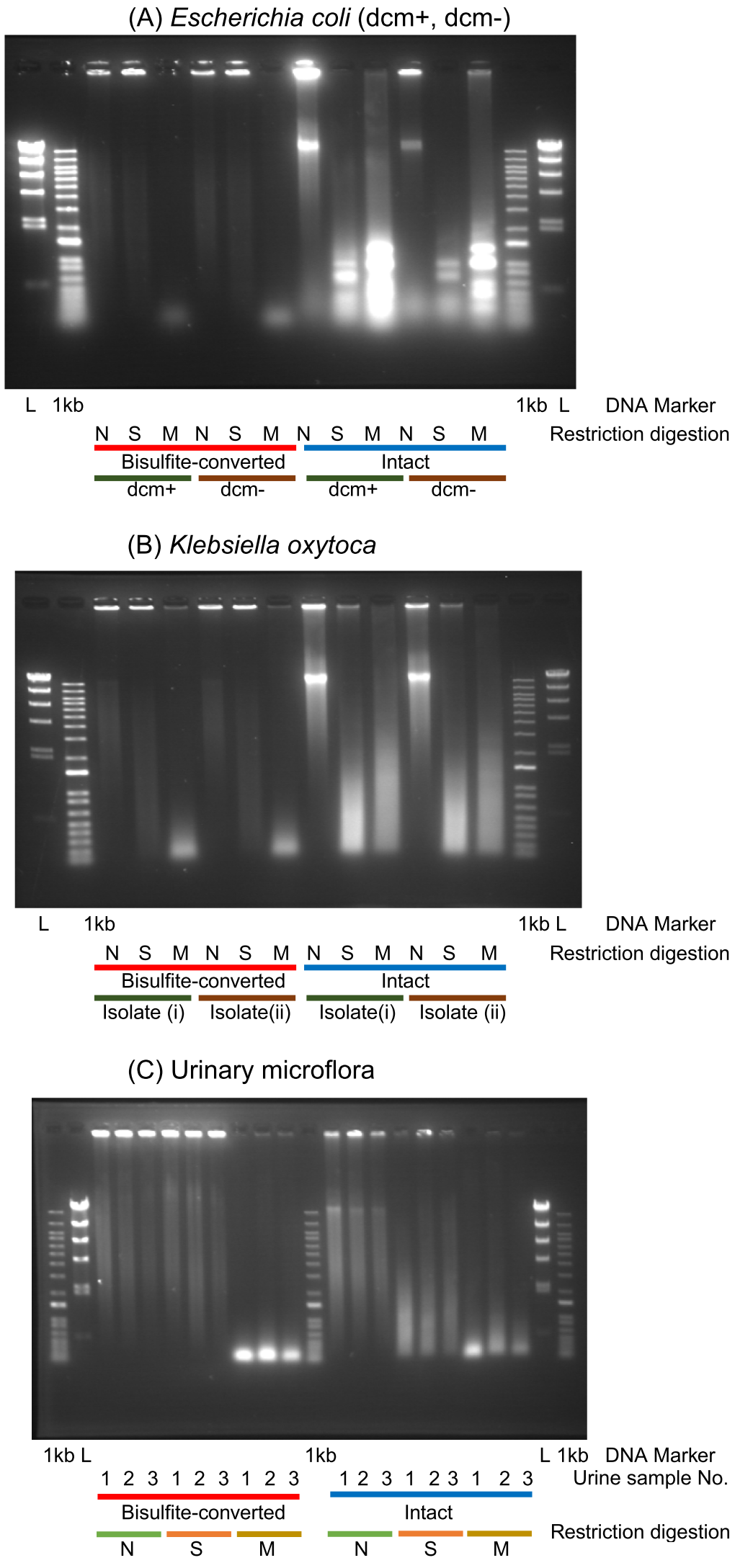


**Supplementary Figure S2**.

**MDA without DNA denaturation (MDAwoDD) successfully amplifies bisulfite-converted DNA**

Bisulfite-converted genomic DNA was amplified with MDAwoDD and analyzed using restriction digestion with Sau3AI or MluCI followed by agarose gel electrophoresis. Intact genomic DNA was amplified with conventional MDA and digested. Because Sau3AI recognizes the 5'-GATC-3' sequence, non-methylated sites are bisulfite-converted and resistant to Sau3AI digestion.

(a) Genomic DNA from *E. coli* (dcm+ and dcm-) was amplified and analyzed. MDAwoDD products from bisulfite-converted DNA were barely digested with Sau3AI but completely digested with MluCI. In contrast, MDA products from intact DNA were completely digested with Sau3AI. These results indicate that most of the MDAwoDD products were derived from bisulfite-converted DNA and that contaminating amplification from undenatured DNA was negligible. There was no discernible difference in digestion patterns between the strains.

(b) Genomic DNA from two clinical isolates of *K. oxytoca* (i and ii) was amplified and analyzed. Bisulfite-converted MDAwoDD products were barely digested with Sau3AI, whereas unconverted MDA products from intact DNA were obviously digested with Sau3AI.

(c) DNA from three clinical urine samples (1, 2, and 3) was amplified and analyzed. Similar to (a) and (b), bisulfite-converted MDAwoDD products were barely digested with Sau3AI, whereas unconverted MDA products from intact DNA were completely digested with Sau3AI. These results indicate that MDAwoDD successfully amplified bisulfite-converted DNA from various samples and that contaminating amplification from undenatured DNA was negligible. L, Lambda/HindIII marker; 100, 100 bp DNA ladder (Takara Bio); N, not digested; S, digested with Sau3AI; M, digested with MluCI.


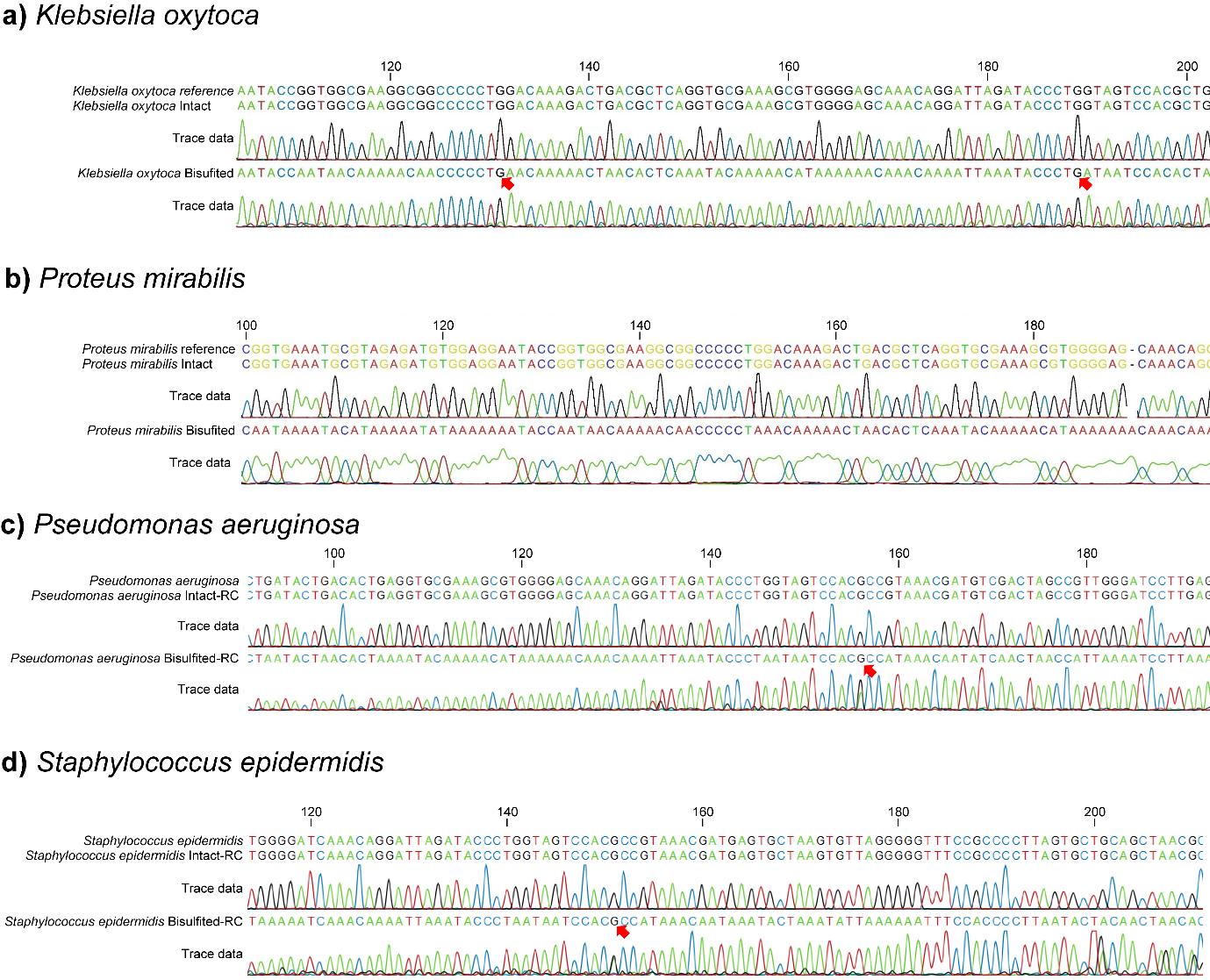


**Supplementary Figure S3**.

**Sequences of sm16S rDNA PCR products from several clinical isolates**

In sm16S rDNA PCR, extracted DNA was bisulfite-treated and pre-amplified with MDAwoDD, while DNA without bisulfite treatment was pre-amplified with conventional MDA. Nested PCR was performed using each MDA product as template and the resulting PCR products were sequenced. Sequences from products without bisulfite treatment (conventional MDA products) were identical to the reference sequences, whereas guanine residues in sequences from the products with bisulfite treatment (bisulfite-converted MDAwoDD products) indicate the existence of methylcytosine residues.

a) Bisulfite Sanger sequencing of a clinical isolate of *K. oxytoca*. Methylation at 5'-CCTGG-3' sites is indicated by the red arrows. The reference sequences were retrieved from NCBI NR_041749.1.

b) Bisulfite Sanger sequencing of a clinical isolate of *P. mirabilis*. 5-Methylcytosine was not detected in the target sequence. The reference sequences were retrieved from NCBI NR_043997.1.

c) Bisulfite Sanger sequencing of a clinical isolate of *P. aeruginosa*. A methylcytosine was detected within the target sequence, as shown by the red arrows towards the guanine residue overlapping with the wave indicating adenine residues. This appears to be a partial DNA methylation pattern, which was detected even after repeating the analysis and changing the primer set. RC means that the presented sequence data is a reverse complement of the raw data. The reference sequences were retrieved from NCBI NR_026078.1.

d) Bisulfite Sanger sequencing of a clinical isolate of *S. epidermidis*. A methylcytosine was detected within the target sequence, as shown by the red arrows towards the guanine residue overlapping with the wave indicating the presence of adenine residues. This appears to be a partial DNA methylation pattern, which was detected even after repeating the analysis and changing the primer set. RC indicates that the presented sequence data is a reverse complement of the raw data. The reference sequences were retrieved from NCBI NR_036904

**
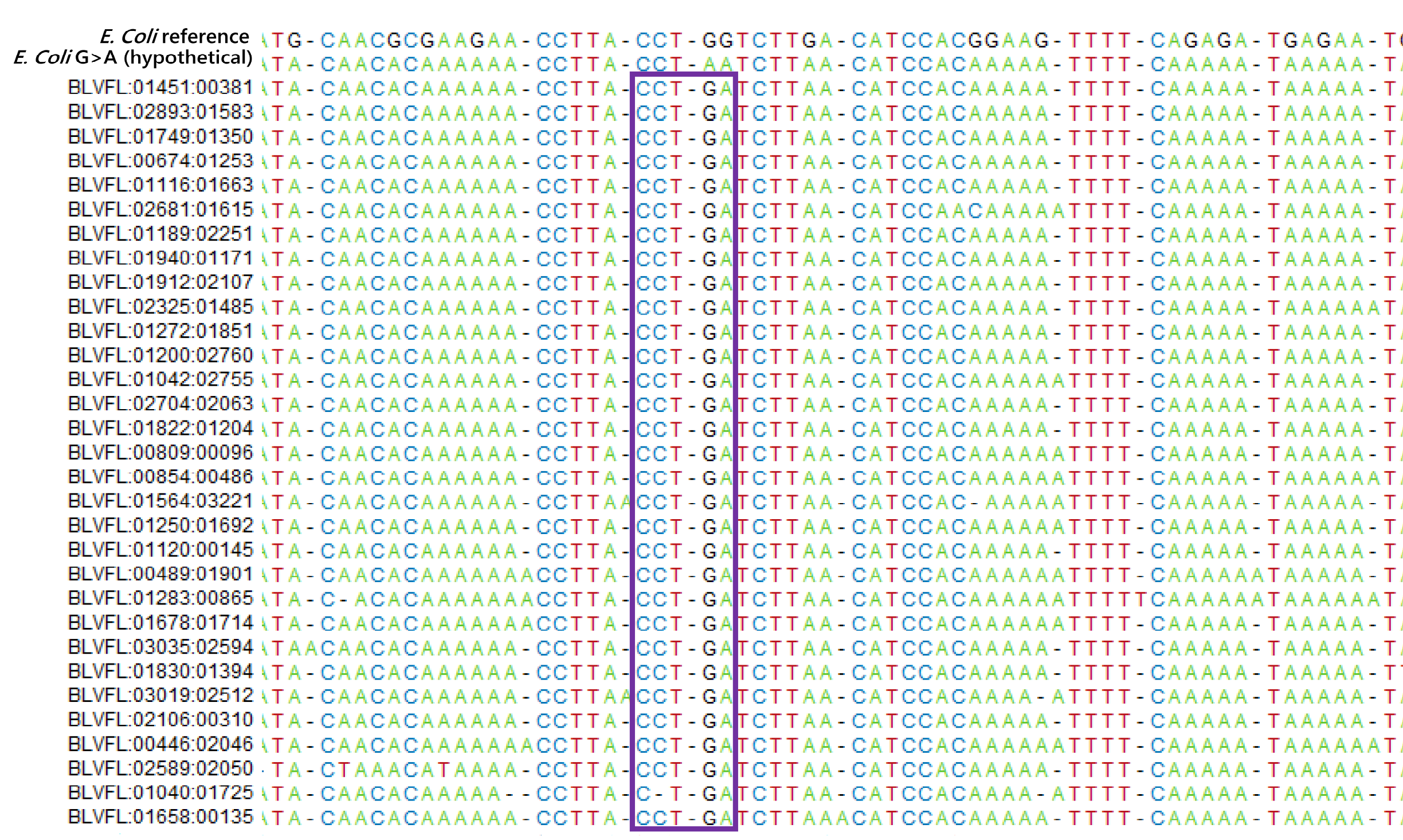
Supplementary Figure S4**.

**Sequence reads implying genomic DNA methylation of *Escherichia coli* in a microflora sample.** The sm16S rDNA sequencing implicated DNA methylation at the CCTGG site (purple boxes) of the genomic DNA fragment that appears to be from *E. coli* in sample number 10_1439. When the cytosine in the negative-sense strand is unmethylated, base G is converted to A, whereas G (black in the purple boxes) in the bisulfite-converted sequences indicates 5-methylcytosine in the negative-sense strand. The reference sequences in the top columns were retrieved from NCBI NC_000913. *Escherichia coli* G>A (hypothetical) sequence was generated by simply substituting nucleotides, assuming that all cytosines are unmethylated and subject to bisulfite conversion. The DNA methylation pattern presented herein may be identical to that of the well-known *E. coli* dcm methylase.


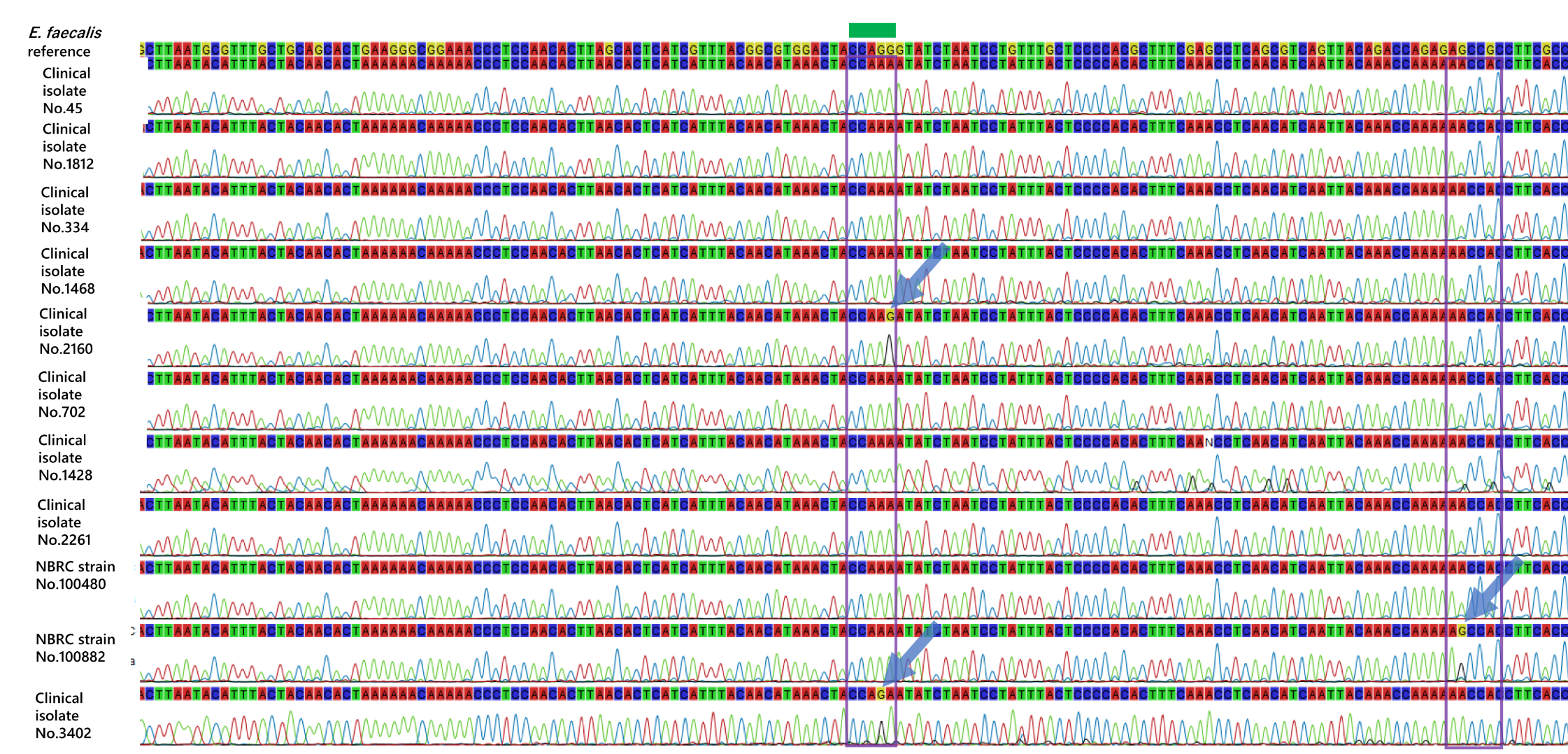


**Supplementary Figure S5**.

**Diversity in stable DNA methylation between *E. faecalis* strains and isolates**

Clinical isolates of *E. faecalis* and NBRC strains were used to verify the DNA methylation status of each isolate and strain. Genomic DNA from nine clinical isolates and two NBRC strains was subjected to sm16S rDNA sequencing. The topmost reference sequence is the intact sequence, not the bisulfite converted. As indicated by arrows, in clinical isolate No. 3402, DNA methylation within the 5'-CC(A/T)GG-3' sequence (overlined in green) was identified; in clinical isolate 2160, methylation occurred in the identical sequence, but here, the end nucleotide is methylated. In NBRC strain 100882, methylation occurred in another sequence, as indicated by the arrow. In the purple boxes, the letters G written in black in the bisulfite-converted sequences indicate 5-methylcytosine in the negative-sense strand.


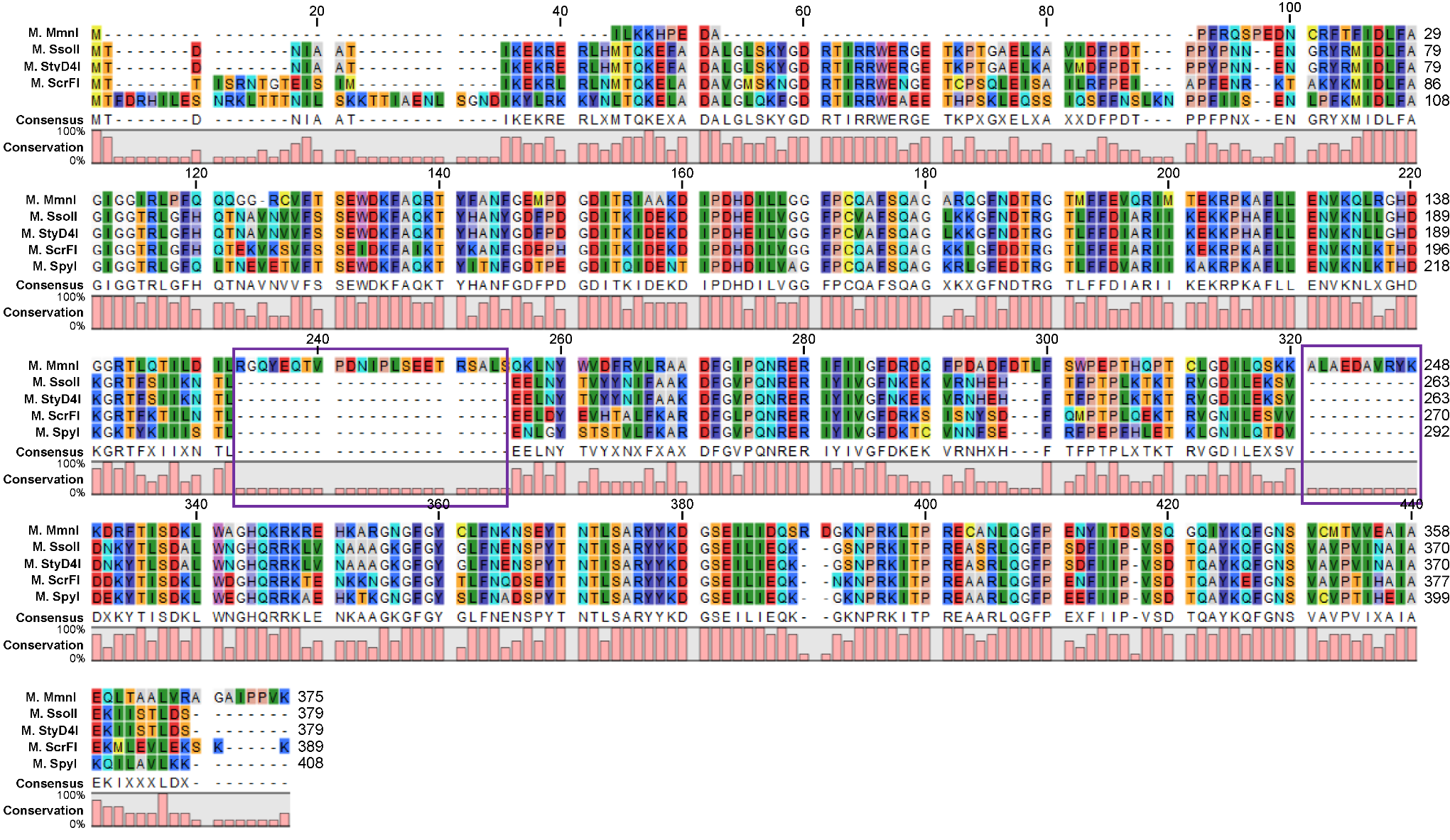


**Supplementary Figure S6**.

**The structure of M. MmnI is distinct from that of M. SpyI**

The amino acid sequence of M. MmnI was compared with that of M. SpyI and closely related DNA methyltransferases from the literature^45^. Although the M. MmnI sequence shares partial homology with that of M. SpyI and other closely related DNA methyltransferases, the M. MmnI sequence exhibits a unique structure, as shown by the amino acid sequence in the purple boxes.


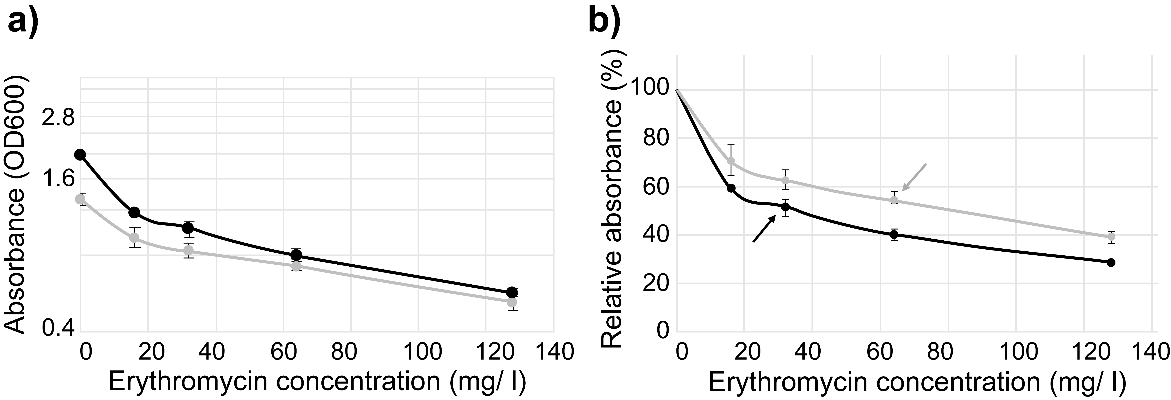


**Supplementary Figure S7**.

**Expression of M. MmnI may be related to erythromycin (EM) resistance**

Four independent cultures of transformed *E. coli* were obtained. The data represent the mean ± standard deviation. a) EM (Merck KGaA, Darmstadt, Germany) was added to liquid LB medium at the indicated concentrations (0, 16, 32, 64, and 128 mg/L). *E. coli* was cultured for 18 h. After culturing, the OD_600_ was measured using the Nanodrop One C (Thermo Fisher Scientific). The results obtained from dcm- *E. coli* transformed with the M. MmnI expression plasmid (pColdIII-MmnI) are plotted with gray lines. Data obtained from dcm- *E. coli* transformed with plasmid lacking the M. MmnI open reading frame (pColdIII-Mor1) are plotted with black lines. The former proliferated more slowly than the latter but seemed to be less affected by EM. b) Estimation of IC_50_ (50 percent growth-inhibitory concentration). The data in a) were replotted using the OD_600_ when 0 mg/L EM was set as 100%. Arrows indicate the observed conditions closest to the IC_50_. The IC_50_ was estimated to be 70 mg/L (with MmnI expression, plotted in gray) and 32 mg/L (without MmnI expression, plotted in black).
